# Reply to Hamidian Jahromi and Bastani: Acute early transplant renal artery thrombosis; a complex etiologic diagnosis

**DOI:** 10.15171/jnp.2016.10

**Published:** 2016-01-02

**Authors:** Mohammad Kazem Fallahzadeh, Neeraj Singh

**Affiliations:** ^1^Department of Internal Medicine, Baylor University Medical Center, Dallas, Texas, USA; ^2^John C. McDonald Regional Transplant Center, Willis-Knighton Health System, and Division of Nephrology, Department of Medicine, Louisiana State University Health Sciences Center-Shreveport, Shreveport, Louisiana, USA

**Keywords:** Kidney transplantation, Renal artery, Thrombectomy, Thrombosis

**Implication for health policy/practice/research/medical education:**

This article discusses renal artery thrombosis as a complication of distal renal artery stenosis in a kidney transplant recipient. This important complication should be considered in the differential diagnosis of acute kidney injury occurring immediately post-kidney transplantation.

## Dear Editor

We read with interest the letter by Hamidian Jahromi and Bastani (1) on our case report (2). The answers to the questions raised in their letter are provided below:

1. Our patient received anti-thymocyte globulin (ATG). There is a case report showing the possible association between OK3 induction therapy and acute transplant renal artery thrombosis (3). There are also reports that show ATG can induce thrombocytopenia, possibly through induction of platelet aggregation (4). However, the chance of local or systemic thrombosis following ATG induction is reported to be minimal (4). Moreover, there is a report that shows rabbit ATG, by induction of thrombocytopenia, can decrease the chance of graft thrombosis in pediatric renal transplant patients (5).
2. Our patient was on aspirin when she was discharged home (6 days post-operation) and at the time of presentation to the hospital with decreased urine output (7 days post-operation).
3. During the catheterization of the transplant renal artery, a complete obstruction was observed and the occluding clot was completely resolved by mechanical thrombectomy and pharmacologic thrombectomy with tissue plasminogen activator (TPA). However, after the removal of the clot, a distal stenosis was visible on follow-up angiogram which was only amenable to stenting. Moreover, fibromuscular dysplasia most commonly has the characteristic string of beads appearance on renal artery angiography (6). This characteristic appearance was not observed in our patient.
4. Heparin infusion was started for our patient following thrombectomy. Activated partial thromboplastin time was within the therapeutic range during the first 24 hours after thrombectomy. Our patient had no episode of hypotension in post-operative period or after the initial attempt at thrombectomy and stent placement.
5. The stent used in our case was Express™ SD Renal and Biliary Premounted Stent by Boston Scientific, Marlborough, MA, USA.
6. All the tests for detection of a possible hypercoagulable state including protein C, protein S and antithrombin III deficiency and mutations of factor V Leiden and prothrombin 20210 were done in our patient after nephrectomy when she became dialysis dependent to see if there is any contraindication for retransplantation. The results of all these tests were negative. Our patient did not have any history of thrombosis in the past. As mentioned in the Discussion part of our case report, renal artery thrombosis in our case was most likely due to distal renal artery stenosis. Although the definitive cause of distal renal artery stenosis in our case was not clear, it was probably due to injury to renal artery during back table preparation of the donor kidney. Renal artery strictures following back table preparation injuries usually take five to seven days to develop.

## Authors’ contribution

MKF and NS drafted the manuscript and approved the final version.

## Conflicts of interest

The authors declared no competing interests.

## Funding/Support

None.
